# Bridging the gap: national virtual education programme for professionals caring for adults with intellectual and developmental disabilities at the time of COVID-19

**DOI:** 10.1192/bjo.2024.67

**Published:** 2024-07-25

**Authors:** Anupam Thakur, Nicole Bobbette, Victoria Bond, Angela Gonzales, Johanna Lake, Gill Lefkowitz, Nadia Mia, Ullanda Niel, Sanjeev Sockalingam, Erica Streisslberger, Kendra Thomson, Tiziana Volpe, Yona Lunsky

**Affiliations:** Adult Neurodevelopmental Services, Centre for Addiction and Mental Health, Toronto, Canada; Queen's University, Kingston, Canada; Surrey Place, Toronto, Canada; Azrieli Neurodevelopmental Centre, Centre for Addiction and Mental Health, Toronto, Canada; Centre for Addiction and Mental Health, Toronto, Canada; Brock University, St Catharines, Canada

**Keywords:** Education and training, intellectual disability, neurodevelopmental disorders, patients and service users, carers

## Abstract

**Background:**

The COVID-19 pandemic significantly impacted the mental health of adults with intellectual and developmental disabilities (IDD). During this period of uncertainty and need for up-to-date information, various virtual training programmes demonstrated the role of tele-mentoring programmes.

**Aim:**

The aim of this paper is to describe the educational evaluation of the National Extension for Community Healthcare Outcomes – Adults with Intellectual and Developmental Disabilities (ECHO-AIDD), a programme for service providers working with adults with IDD during COVID-19.

**Method:**

The programme consisted of six sessions, conducted weekly, over two cycles. Each session included didactic teaching by hub team members, COVID-19 news updates, wellness check-ins and a brief mindfulness activity, followed by a 30 to 45 min case-based discussion. The hub structure had an inter-professional approach to team expertise. Those with lived experience were an integral part of the content experts’ hub. Pre-, post- and follow-up evaluation data were collected.

**Results:**

Care providers from health and social care sectors (*n* = 230) participated in the programme. High levels of engagement and satisfaction were reported. Self-efficacy ratings improved from pre- to post-, and were maintained at 8-week follow-up; improvement from pre- to post- was significant (*P* < 0.0001).

**Conclusion:**

Exposure to National ECHO-AIDD educational intervention led to improvement in perceived competencies. This study also shows the valuable role of people with lived experience in fostering adaptive expertise in learners. The outreach and scalability support the feasibility of building a national virtual community of practice for IDD service providers. Future studies should focus on studying the impact of these programmes on the health outcomes of people with IDD.

The COVID-19 pandemic significantly impacted the mental health of adults with intellectual and developmental disabilities (IDD).^1,7^ Adults with IDD were disproportionately affected by pandemic-related stressors including public health restrictions, abrupt disruption in daily routines and changes in community supports.^8,9^ As the focus shifted on emergence from the pandemic, an exponential increase in these stressors, compounded by a surge in challenging behaviours, mental health issues and overprescribing of psychotropic medications,^5,10^ continues to challenge healthcare providers to deliver support for this vulnerable population. Though the pandemic waned, the health disparities that decrease life expectancy in the IDD population remain. Service providers need to be equipped with the necessary skills and training to bridge this gap, and ensure high-quality healthcare for adults with IDD. Building capacity in the community was of critical importance during the pandemic. Concerted efforts from service providers in the social service and health sectors were needed to address the mental health needs of adults with IDD.^5^ Various virtual training programmes, such as the Project Extension for Community Health Outcomes (Project ECHO) demonstrated the role tele-mentoring could play, by sharing best practices and creating virtual communities of practice.^5,11,14^ Project ECHO is a ‘hub and spoke’ tele-educational capacity building model that leverages the use of video-conferencing technology for enhancement of learner knowledge and skills.^15^ The ‘hub’ consists of the expert team, and participants in satellite sites are referred to as ‘spokes’. It follows an ‘all teach, all learn’ philosophy. In addition to the need to equip service providers with the skills to support the mental health of adults with IDD,^16^ experience from previous pandemics and early findings from the COVID-19 pandemic highlighted the importance of service providers’ mental health.^17,18^ Service providers working with adults with IDD reported high levels of stress in their workplace during the pandemic.^18,19^ Tele-education played an important role in supporting the mental health needs of service providers during the pandemic.^14,20,21^ Thakur et al^14^ reported on the design and delivery of a provincial virtual education programme, ‘ECHO Ontario adult intellectual and developmental disabilities: mental health in the time of COVID-19’, using this model to support service providers caring for adults with IDD. The innovations in the programme include curated COVID-19-specific information, self-care strategies, wellness checks, mindfulness sessions for participants, and family perspective in the programme. The curriculum was co-produced and co-delivered with a family caregiver hub member. High levels of engagement, self-efficacy and a sense of being part of a community of practice (CoP) were reported in this virtual learning programme. However, the pilot programme was limited to one province in Canada, it did not include anyone with IDD on the teaching team, and the longer-term impact of the programme was not studied.

The aim of this paper is to describe the educational evaluation of the National Extension for Community Healthcare Outcomes – Adults with Intellectual and Developmental Disabilities (ECHO-AIDD), a nationwide programme for service providers working with adults with IDD, during COVID-19. The course content remained similar to the regional programme described in a previous paper,^14^ and the teaching team was expanded to include an adult with IDD. The evaluation of the programme included an additional follow-up time point of 8 weeks after the course was completed.

## Method

This project was part of a broader effort to build the capacity of adults with IDD, family caregivers, and health and social service providers from across Canada to address mental health concerns during the pandemic.^22,23^ The ECHO-AIDD programme was one of three parallel courses co-designed and delivered with clinicians and adults with IDD and/or family caregivers.

### ECHO-AIDD (Canada)

#### Programme and setting

The goal of this Canada-wide virtual training programme was to build service provider capacity in supporting the mental health needs of adults with IDD during COVID-19. Skill enhancement and psychological well-being of participants were the focus of the programme. Similar to the previous pilot programme, each session included didactic teaching by hub team members, COVID-19 news updates, wellness check-ins and a brief mindfulness activity, followed by a 30 to 45 min case-based discussion. The didactic curriculum topics were: (1) COVID-19 overview; (2) mood, anxiety and suicide risk assessment; (3) health promotion through integrated care planning and communication; (4) grief and loss; (5) supporting families and family interventions; and (6) self-care and wellness. The topics were retained from the previous programme because of their continued relevance and high levels of satisfaction and self-efficacy, as per the previous programme evaluation data.^14^ An anonymised case was presented by a service provider participant (a ‘spoke’) for which they required support. The selection of cases highlighted the complexity in caring for adults with IDD at the time of COVID-19. A case form was used by the case presenter to record clinical history, including de-identified data on current problems, past psychiatric and medical history, medications, lived experiences, support plans and specific questions, for which they requested the opinion of hub and spoke members. Case discussions had an inter-professional education focus, including people with lived experience. Details of the course design are described in a previous publication.^14^

#### Participants

Participation was open to all service providers working with adults with IDD, in both health and social care. Potential participants were recruited by email and social media, sent via national and provincial organisations involved in the research study, including community IDD agencies, mental health service providers and professional organisations, as well as by sharing information with previous participants of ECHO programmes at the Centre of Addiction and Mental Health in Canada. Interested participants (spokes) completed a web-based programme registration form, and a Statement of Collaboration which outlined expectations for participation in the programme, including attending at least 60% of sessions, presenting cases for discussion and participating in programme evaluation. An online orientation session was offered to familiarise participants with the video conferencing tools, and to check whether they needed any accommodation or support.

Hub members included a psychiatrist, psychologist, nurse, doctoral level-certified behavioural analyst, social worker, occupational therapist, family caregiver and an adult with IDD. The hub structure reflected an inter-professional collaborative approach to team expertise. Those with lived experience were an integral part of the content experts’ hub, actively involved in curriculum design, creation and delivery, and their unique lens contributed to understanding and addressing complex clinical problems. Support from an operations team helped to resolve any technical issues encountered in the sessions, such as audio or camera issues, difficulty connecting and/or screen-sharing didactic materials.

The programme consisted of six sessions, each lasting 1.5 h, conducted weekly over two cycles from November 2020 to March 2021. The sessions were conducted using Zoom video conferencing technology. Following each session, hub members reviewed participants’ feedback from the previous week, which facilitated improvements to meet participants’ needs. The timing of the programme was chosen after careful consideration of the convenience of participants joining across six time zones. All course registrants were invited to participate in a research evaluation, but it was not a requirement for course participation. Our team wanted to prioritise the sharing of urgently needed information over research involvement, recognising the time limitations and stress of that time-period. Those who opted for the research were eligible to take part in a raffle for gift cards or learning materials.

### Evaluation measures and outcomes

#### Design

The programme was reviewed and approved by the Institutional Research Ethics Review Board (REB No 123-2020). Programme evaluation of ECHO-AIDD (Canada) was informed by the first five levels of Moore's evaluation framework (participation, satisfaction, learning, self-efficacy and performance).^24^ Other ECHO programmes used this framework to evaluate outcomes.^25,26^ Informed consent was obtained from all participants. Consenting course participants completed outcome measures at three time points: pre- (1 week before the course), post- (1 week after the course) and follow-up (8 weeks following the completion of the course).

#### Measures and data collection

Pre-, post- and follow-up data were collected using REDCap (version 14.0.16, REDCap Research platform). Survey measures were supplemented by additional questions related to the experiences during the COVID-19 pandemic.

#### Participation (level 1)

Demographic information of participants, including professional background and weekly attendance, was collected for the duration of the programme.

#### Satisfaction (level 2)

Participant satisfaction was assessed using online surveys which were sent out following each session. A time frame of 1 week was set for completing the surveys. A 5-point Likert scale from 1 (strongly disagree) to 5 (strongly agree) was used to rate statements, with a focus on expansion of knowledge and skills, reduction in professional isolation, learning needs being addressed, session recommendation to others and overall satisfaction. Additional post measures were used to evaluate satisfaction with the number of participants in the course, learning environment (inter-professional health and social care providers), family member and adult with IDD as hub content experts, mindfulness activity, curated COVID-19 updates and feeling supported in a virtual CoP.

#### Learning and competency (levels 3 and 4)

Self-efficacy was assessed for four core programme competencies: (1) ‘I am confident in my ability to communicate effectively and prepare for person- and family-centred care for adults with IDD during the COVID-19 pandemic (SE1)’, (2) ‘I am confident in my ability to support and manage the mental health of individuals with or suspected of having IDD during the COVID-19 pandemic (SE2)’, (3) ‘I am confident in my ability to appropriately manage burnout and build resilience in myself, other healthcare and social service providers, and caregivers during the COVID-19 pandemic (SE3)’ and (4) ‘I am confident in my ability to work effectively in/with inter-professional and intra-professional teams across health and social systems during the COVID-19 pandemic, to support the care of clients with IDD (SE4)’. A previously established 4-item rating scale with 0–100-point confidence range was used to rate self-efficacy related to caring for adults with IDD during the pandemic, before and after the programme, and at follow-up (8 weeks), described in Table 4. Higher numbers were indicative of higher confidence. Competencies were developed by the hub team, informed by previous ECHO-AIDD programmes, a review of literature and consensus among experts.

#### Changes in practice (level 5)

Practice change was assessed at course completion and at 8-week follow-up. Participants responded whether the programme resulted in a change in their practice, using a binary scale (yes/no), supplemented by open-text feedback to provide examples.

Additional items related to programme feedback, professional support and coping with stressors during COVID-19 were included in the evaluation. At follow-up, participants were asked to reflect on the strategies or tools they incorporated in their professional and personal life since attending the course.

### Data analysis

Quantitative data were analysed using a combination of SPSS software (version 21, IBM Corp) and Microsoft Excel. Frequencies and percentages were collected for categorical variables, and means and standard deviations were calculated for the continuous variables. Pre-, post- and follow-up data were analysed using repeated measure ANOVA with Greenhouse-Geisser correction. Bonferroni adjustment was used for post-hoc analysis. Statistical tests were 2-sided, with a significance set at level of 0.05%.

Open-text responses from the ECHO post-cycle surveys were analysed using inductive thematic analysis.^27^ This approach facilitated the reduction of large amounts of qualitative data into meaningful themes and conclusions. A member of the research team (V.B.) read all open-text responses, identifying preliminary themes present across the surveys. Project team members (Y.L. and N.B.) reviewed and refined the coding process. Themes were solidified and defined, while exemplary quotes were identified for each theme.

## Results

### Participation

A total of 230 service providers, distributed across the country, signed up for the ECHO-AIDD (Canada) programme, and 102 of them, from diverse professional backgrounds and different provinces of the country, consented to participate in the research evaluation (Table 1, Table 2). Of those, 75 participants completed the pre and post surveys, and 61 completed surveys at all three time points (pre-, post- and follow-up). Out of 102 learners, 81 attended more than four sessions, and 44 attended all six sessions. Participants were mainly social care providers, case workers or case managers, behavioural therapists/analysts and social workers (see Table 1).

**Table 1:** Participant distribution by profession and practice setting

Demographic group	Value, *n* (%)
Participants by profession	102 (100)
Behaviour analyst/behaviour therapist	11 (11)
Case worker/case manager	19 (19)
Developmental service worker	33 (32)
Nursing professional	3 (3)
Occupational therapist	1 (1)
Physician	4 (4)
Psychotherapist	1 (1)
Social worker	8 (8)
Other^a^	22 (21)
Organisations by practice setting	82 (100)
Community or group homes, supported living agencies	35 (43)
Community-based organisation/community office/client home	21 (25)
Consulting to community organisations, health providers or private practice	8 (10)
Employment setting or vocational centre	4 (5)
Long-term care	3 (4)
Mental health organisation	1 (1)
Paediatrics	1 (1)
Residential setting	5 (6)
Social services	4 (5)

a. Autism employment consultant, human service counsellor, care home operator, programme coordinator, group home supervisor, community developer.

**Table 2:** Breakdown of participants by province/territory

Province/territory	No. of participants in cycles 1 and 2, *n* (%)
Alberta	18 (17.5)
British Columbia	8 (8)
Manitoba	5 (5)
New Brunswick	4 (4)
Newfoundland and Labrador	10 (10)
Nova Scotia	6 (6)
Ontario	29 (28)
Prince Edward Island	1
Saskatchewan	21 (20.5)

There were no participants from Quebec, Nunavut and Yukon.

### Satisfaction

A total of 176 weekly satisfaction surveys were completed across the two cycles, with an average survey response rate of 22 participants. High mean ratings were observed in all the satisfaction domains, ranging from 4.07 (0.13) to 4.43 (0.08) (see Table 3). Overall mean satisfaction with the sessions was 4.36 (0.08). Out of 75 respondents who completed the end of programme evaluation, 87% agreed that their expectations were met through participation in the ECHO-AIDD (Canada) programme.

**Table 3:** ECHO-AIDD (Canada) participant weekly satisfaction ratings

Statement	Rating out of 5 (*N* = 176), mean (s.d.)
The session content has expanded existing skills and knowledge	4.07 (0.13)
The session has addressed my learning needs	4.2 (0.24)
The session has reduced my professional isolation	4.2 (0.05)
I would recommend this session to others	4.43 (0.08)
Overall, I was satisfied with the session	4.36 (0.08)

### Learning and competency

Sixty-one participants completed surveys at pre-, post- and follow-up which were considered for further analysis. An analysis of the four competencies separately showed a statistically significant improvement in self-efficacy ratings from pre- to post-, and from pre- to follow-up for each item (see Table 4). The majority of respondents agreed that having adults with IDD (95.74%) and family members (93.62%) as faculty members enhanced their learning. Also, most (98.68%) agreed that inter-professional health and social care providers learning together enriched their learning.

**Table 4:** ECHO-AIDD (Canada) competencies and self-efficacy (SE) scores

Competency	Pre mean (s.d.)	Post mean (s.d.)	Follow-up (8 weeks) mean (s.d.)
I am confident in my ability to communicate effectively and prepare for person- and family-centred care for adults with IDD during the COVID-19 pandemic (SE1)	69.02 (17.97)	80.97 (13.41)	80.54 (14.06)
I am confident in my ability to support and manage the mental health of individuals with or suspected of having IDD during the COVID-19 pandemic (SE2)	62.23 (21.90)	75.82 (16.41)	76.03 (16.65)
I am confident in my ability to appropriately manage burnout and build resilience in myself, other healthcare and social service providers, and caregivers during the COVID-19 pandemic (SE3)	60.74 (19.03)	71.97 (18.43)	71.49 (18.54)
I am confident in my ability to work effectively in/with inter-professional and intra-professional teams across health and social systems during the COVID-19 pandemic to support the care of clients with IDD (SE4)	73.54 (18.18)	81.56 (13.98)	81.75 (15.33)
Mean self-efficacy ratings (SE)	66.38 (15.62)	77.58 (13.39)	77.45 (13.54)

Post hoc analysis with a Bonferroni adjustment revealed that self-efficacy ratings increased significantly from pre- to post- for SE1 (*P* < 0.0001), SE2 (*P* < 0.001), SE3 (*P* < 0.001), SE4 (*P* < 0.05 and mean self-efficacy (*P* < 0.01). Similarly, self-efficacy ratings increased significantly from pre- to follow-up for SE1 (*P* < 0.001), SE2 (*P* < 0.001), SE3 (*P* < 0.01), SE4 (*P* < 0.05) and mean self-efficacy (*P* < 0.001)

IDD, intellectual and developmental disabilities.

### Practice change (post course and at 8-week follow-up)

More than half of the 75 respondents (*N* = 39, 52%) agreed that participation in ECHO-AIDD (Canada) resulted in a change of practice at the end of the course. At that point, participants (*N* = 75) were less stressed at work (pre = 3.68, post = 3.43, *P* < 0.005), were more accepting of the risk of caring for COVID-19 patients (pre = 3.56, post = 3.79, *P* < 0.05) and less worried about not working because of COVID-19 (pre = 2.07, post = 1.87, *P* < 0.05), all at significant levels. Further to participation in the course, learners reported having ‘enough professional support and resources for themselves, to continue caring for clients’, and ‘felt equipped to cope with stressors related to the pandemic’ (i.e. fear of contagion, rapid spread of virus, risk to family and friends). Notably, there was a trend for some improvement in being supported professionally at 8 weeks, but this was not maintained at follow-up (Table 5). Similarly, the scores for feeling equipped to cope with COVID-19 stressors (pre = 3.31, post = 3.84, follow-up at eight weeks = 3.57) were significantly higher from pre-scores (*P* < 0.001) and at post-evaluation, but not maintained at follow-up.

**Table 5:** Changes in perceived COVID-19 support following course participation

Measure	(I) Time	(J) Time	Mean difference (I – J)	Std error	Significance^b^	95% CI for difference	
I feel I have enough professional support and resources for myself to continue caring for my clients during this time	1	2	−0.328	0.134	0.051	−0.657	0.001
		3	−0.131	0.103	0.626	−0.385	0.123
	2	1	0.328	0.134	0.051	−0.001	0.657
		3	0.197	0.140	0.495	−0.148	0.541
	3	1	0.131	0.103	0.626	0.123	0.385
		2	−0.197	0.140	0.495	−0.541	0.148
I feel I have enough professional support and resources for myself to continue caring for my clients during this time	1	2	−0.525^a^	0.121	0.000	−0.822	0.228
		3	−0.262	0.109	0.059	−0.532	0.007
	2	1	0.525^a^	0.121	0.000	0.228	0.822
		3	0.262	0.132	0.155	−0.063	0.587
	3	1	0.262	0.109	0.059	−0.007	0.532
		2	−0.262	0.132	0.155	−0.587	0.063

Based on estimated marginal means.

1 – pre-, 2 – post-, 3 – follow-up at 8 weeks.

a. The mean difference is significant at the 0.05 level.

b. Bonferroni adjustment for multiple comparisons.

Six themes were identified from the post-evaluation data, and this knowledge helped to provide additional insights into the experience and perceived value of the programme.

#### Theme 1: tools and resources

Participants found the tools and resources shared in the course useful in supporting the needs of adults with IDD in their practice. From the perspective of one participant, ‘having information and access to visual tools to share that information has been excellent for teaching and learning’. Furthermore, a participant noted, *‘*It has given me better tools to support my staff team and people supported during this time’. The extensive list of ideas and validation of approaches and interventions was reported to be especially valued by participants, as one participant noted: ‘I was glad to have the CAMH resources and resource people at hand to help me focus on staying hopeful. The individuals I work for benefited from my participation because I shared my learnings with them’. The Health, Environment, Lived experience and Psychiatric conditions (HELP) framework^28^ in particular was identified as a helpful tool, and many participants reported using the HELP framework in the treatment plans.

#### Theme 2: community of practice

Participants reflected on feeling ‘a sense of community’, and how it made them feel supported and empowered as individuals. Project ECHO-AIDD ‘allowed a space of like-minded professionals to come together and discuss the challenges we face in a positive and problem-solving way’, as one participant noted. Participants also spoke to the sense of community this programme created among participants: ‘participating in this ECHO program has made me feel a sense of community, and broadened my awareness of what other people in roles that are similar to mine are going through. It informed me that there are many resources available and that I am not alone’. The sense of community also functioned to address professional isolation that many participants felt, for example as one participant reported: ‘The ECHO program has allowed me to feel less alone in my profession as many other professionals were experiencing similar challenges all with the same goal of wanting to help individuals/families’.

#### Theme 3: national perspective of the programme as the ‘highlight’

Participants specifically highlighted the value of a national perspective and reflected on the benefits of connecting with other service providers across the country. As one participant noted, ‘I like that it was Canada-wide and able to learn how all provinces were affected. Don't feel as secluded’. Furthermore, participants reported that it was ‘helpful to connect with others across the country and hear about resources that I was not familiar with’. One respondent noted that while acknowledging the different service contexts and teams in Canada, ‘it was great to hear from other people across the country that are dealing with similar issues. All the input and feedback from people with different backgrounds and expertise was very helpful’.

#### Theme 4: wellness: self-care and caring for team members

Service providers valued the opportunity to reflect on caring for themselves and others. One participant noted that for themselves, ‘having the mindset session at the beginning was a great part of taking time for your own personal care and mental health’. Also, others noted that they used it as a tool to promote psychological well-being in their workplace, for example, they ‘create[d] a monthly check-in with my team to ensure they are emotionally ok [and](not feeling isolated)’ and participants were ‘able to utilise the information for employee burnout within [their] team’. As well, participation in the mindfulness sessions of the programme was particularly valued and it promoted ‘an even greater appreciation and understanding of empathy, communication, self-care. Taking a moment to breathe – using this approach with my clients and also [themselves]’. Many participants noted that ‘mindfulness has been very helpful. Practising self-care [and] reaching out for support through outside sources and other organisations’.

#### Theme 5: involvement of adults with IDD and family members

Participants highlighted the impact of hearing from individuals with lived experience during the course, citing that the inclusion of adults with IDD and family caregivers as teachers broadened their understanding and contributed to interdisciplinary learning. As one participant noted, ‘the family and self-advocate has given me even a greater appreciation for the difficulties they encounter every day and especially during these times’. Another participant reported that ‘it was also really nice to see perspectives from family members and persons with lived experiences; a good reminder to be mindful of their lenses’. Overall, there was an appreciation for ‘different professionals and people with lived experience’. This participant further explained ‘that sometimes [they felt] that the medical model is rather siloed and unresponsive to the wisdom of patients and other professionals. Interdisciplinary learning as well as learning from clients is really important’.

#### Theme 6: programme participation and implementation of new learnings

Participants reflected on several factors that facilitated their participation in the course, including the convenience of virtual sessions, course duration and time, workplace support to attend and technological assistance from the hub. Although most did not encounter any significant difficulties attending the course, some participants reported barriers such as clinical duties, (lack of) time and technological challenges (e.g. internet connectivity issues). Lack of time was a common reason for participants who missed sessions. Regarding implementation of the virtual education from the programme and overall practice changes, participants noted the following: they applied their learning to improve client and family engagement, used tools to enhance health communication with client and health providers, shared patient and family-oriented tools about COVID-19 and implemented wellness initiatives. Participants’ reflections on practice change at the 8-week follow-up after the course are further summarised in Table 6.

**Table 6:** Practice change reflections by participants at 8-week follow-up

Content theme	Illustrative quotes
Applied their learning to improve engagement with clients and families	‘I am listening more intently, being present and affirming residents, family and colleagues, paying closer attention to detail and asking what is the person not saying.’ ‘I have become more open to actively listen to the fears and concerns, not dismiss them, but to work through the fears with the individual and look at ways to alleviate those fears in a positive and productive manner.’
Used tools to enhance health communication with their clients and health providers	‘HELP framework as a way of understanding and addressing health issues.’
Shared patient- and family-oriented tools about COVID-19	‘Sharing patient- and family-oriented tools about COVID and about vaccines with clients and families from the H-CARDD COVID site.’ ‘We do a COVID-19 update every week (just as the course did), we have created an internal weekly course that provides information and coping strategies for our individuals.’ ‘I have worked directly with provincial health services to create a “coping with COVID” webinar for individuals across the province.’
Wellness initiatives	‘I now do mindful moments with clients and the start of check-in meetings to model the behaviour I am asking them to try out.’ ‘I have connected with another person who participated [in ECHO] for support.’

ECHO, Extension for Community Health Outcomes; H-CARDD, Health Care Access Research and Developmental Disabilities; HELP, Health, Environment, Lived experience and Psychiatric conditions.

## Discussion

### National ECHO-AIDD programme

We describe the successful implementation of a Canada-wide ECHO-AIDD programme to improve the self-efficacy and well-being of health and social care providers in caring for the mental health of adults with IDD during the COVID-19 pandemic. This pre-, post- and follow-up evaluation study demonstrates that exposure to the ECHO-AIDD educational intervention led to improvement in perceived competencies, which was maintained at 8-week follow-up. High engagement, satisfaction and retention rates suggest that the ECHO-AIDD programme, like many other ECHO programmes,^29,31^ was an effective model to share best practices and improve provider skills during the pandemic. The scalability at a national level during the COVID-19 pandemic was a highlight of the programme and indicates the feasibility of such capacity-building programmes for the future. Notably, the qualitative feedback at follow-up suggests participants continued to integrate important aspects of the course, such as the tools and self-care care strategies, in their professional and personal lives after attending the course. This demonstrates the effectiveness of the course and its promise for the future in the post-pandemic era.

### ‘Experts by experience’ and adaptive expertise

People with lived experience (an adult with IDD and a family caregiver) were ‘experts by experience’ (EBE)^32^ in the ECHO-AIDD programme. Embedding EBE in the co-production and co-delivery was a key aspect of the programme. Their contribution supported learners in the development of adaptive expertise in the domains of integrated understanding of clinical complexities, collaborative reformulation and generation of creative new solutions. This type of case-based learning approach to develop mastery in complexity has also been described in previous ECHO programmes^11^ and residency programmes.^33^ This study describes the valuable role of people with lived experience in fostering adaptive expertise in learners. Participants valued the opportunities to learn from those with lived experience, which led to evolution, refinement and development of knowledge and skills of the participants to practise more effectively. Involvement of people with lived experience as teachers required all members of the hub team to act with sensitivity and to be aware of potential issues of power dynamics and tokenism.^34^ In addition to valuing their important role as teachers, there is a need to invest in providing appropriate support to ensure their success. It is important to make such teaching experiences more accessible and equitable. Strategies such as having more than one family member or adult with IDD on the teaching team to reduce burden and expectations, provide appropriate compensation and regular check-ins or debriefs with a designated team member can be helpful. Educators and programme leaders can use a Health Equity and Inclusion Framework^35^ to create equitable and inclusive education and training programmes.

### Virtual CoP

As a part of the CoP, participants appreciated the opportunity to relate to colleagues across the country and learn from each other. Similar to the provincial programme,^14^ the availability of COVID-19 related resources and self-care support for service providers was beneficial. Participants shared these strategies both with clients and within their teams, demonstrating the role of the CoP in facilitating knowledge mobilisation and dissemination. One of the participants commented on the impact of the ECHO-AIDD CoP and summed it up as ‘Many hands make light work’, reflecting on the collective teaching and learning created through the ECHO and encapsulating the CoP spirit. Given the impact of COVID-19 on the mental health of service providers and feelings of loneliness,^19,36^ participants reflected on how the ECHO-AIDD CoP helped reduce professional isolation. The sessions on wellness and wellness strategies offered service providers a greater understanding of their own self-care, which had an impact beyond the ECHO-AIDD programme.

The outreach and scalability of using the ECHO model supports the feasibility of building a national virtual CoP for IDD service providers, connecting geographically dispersed areas. Similar to the principles identified in previous studies,^34,37^ several factors in the ECHO-AIDD programme contributed to its success as a CoP. These include clear objectives, strong sponsorship, a dedicated operations team for technological support, prioritisation of participant safety and built-in evaluation methods for outcome measurement. This programme is a part of the broader ECHO programme, benefiting from a robust implementation framework.^38^

Although this study had many strengths, there are also several limitations. Not all people who participated in the programme completed the research evaluation, and it is possible that there were differences in the experiences of those who opted not to complete research measures. Future research, during less intensive times, could make research participation a requirement so that people who drop out can be compared to people who complete the programme. In addition, future programme evaluation could include follow-up of participants who did not complete all the ECHO sessions or did not participate in the evaluation, to determine the causes and explore if there were any differences from those who participated in all the sessions. Additional incentives (honoraria instead of a raffle prize) could be important in future research, given the heavy demands faced by service providers. The absence of a control group makes it hard to know which changes were related to the intervention as opposed to the changing context of the pandemic or other potential supports they were receiving. For example, the pandemic situation worsened as the courses were ending. Again, given the urgent nature of the pandemic, we did not think it was ethical to deny participation to anyone by being in a control group, but it would be important in future research. While the course content focused largely on mental health, there were several physical health issues of concern raised by course participants, including the vaccination rollout. Although this was a topic of discussion as it emerged, there was no formal evaluation of the impact of the teaching and support on vaccination strategies.

Overall, results demonstrate that the virtual educational model can be utilised in an inter-professional context for a diverse group of learners, transcending geographical barriers. The ECHO-AIDD programme is a promising virtual education model for future IDD training initiatives. Future programmes should consider extending areas of practice to specific IDD populations and concerns, for example, autism and ageing in IDD. It will also be important to study the impact of these programmes on the health outcomes of people with IDD. As the pandemic waned, an increased intent to change practice and the retention of learning underscored the programme's relevance in building clinical capacity, bridging sectors and closing the gap in access to high-quality healthcare in the longer term.

## Data Availability

The data that support the findings of this study are available from the corresponding author, A.T., on reasonable request.
